# Atypical and isolated posterior reversible encephalopathy syndrome in postpartum without preeclampsia: a case report

**DOI:** 10.11604/pamj.2023.44.68.34253

**Published:** 2023-02-03

**Authors:** Houda Moustaide, Karima Aouali, Saad Benkirane

**Affiliations:** 1 Department of Gynecology and Obstetrics, Faculty of Medicine, Abdelmalek Essaadi University Hospital, Tangier, Morocco

**Keywords:** PRES syndrome, preeclampsia, seizure, maternal health, case report

## Abstract

Posterior reversible encephalopathy syndrome (PRES) is a neurological complication frequently found during brain exploration for severe preeclampsia when it is associated with neurological signs. Being a newly discovered entity, its mechanism of genesis is still based on a hypothesis not yet confirmed. The clinical case that we report highlights an atypical PRES syndrome occurring in postpartum without any signs of preeclampsia. The patient had suffered a state of convulsive dysfunction after delivery without hypertension and the diagnosis of PRES syndrome was confirmed based on the results of the brain computed tomography (CT) scan, she showed signs of clinical improvement on the fifth day of postpartum. Our case report calls into question the association between PRES syndrome and preeclampsia that we find in literature and puts a big question mark on the causal link between the two in pregnant women.

## Introduction

Posterior Reversible Encephalopathy Syndrome (PRES) is a radiological expression of vasogenic cerebral edema that clinically manifests as a multitude of neurological symptoms. This is a newly described entity that appears directly in the brain CT scan but its mechanism is still poorly understood. However, during pregnancy, PRES syndrome often occurs with severe preeclampsia or eclampsia; hence, the great interest of our clinical case, through which we report the case of an isolated PRES syndrome occurring in postpartum and outside the context of preeclampsia.

## Patient and observation

**The patient information:** our patient was a 21-year-old primiparous with no particular history; not followed for any neurological pathology neither autoimmune nor antecedents of transplantation of organs and without notion of particular drug taking.

**Clinical findings:** our patient was admitted for childbirth which was said to be at term. The general examination on admission found a conscious parturient well oriented in time and space, blood pressure at 10/5 cmHg, labtix was negative without neurosensory signs of hypertension in particular no headache or visual disturbances, absence of edemas in lower limbs. Obstetric examination: uterine height was normal at 31 cm with a flexible abdomen and absence of contracture, the patient was in the active phase of labor with a dilation of 5 cm, the presentation was cephalic, and a clinically normal pelvis, the water bag was ruptured with clear fluid and no bleeding. Neurologic examination was unremarkable with normal tendon reflexes. An initial laboratory test showed a correct hemoglobin level at 12.6 g/dl and a platelet count at 221,000 el/mm^3^; the crase balance was normal and the O^+^ grouping. The evolution of work was harmonious; giving vaginal birth to a female newborn with a birth weight of 3000g Apgar at 10/10 aspect at term, delivery was directed and examination of the delivered was normal and complete. In the immediate postpartum period, the patient's hemodynamic state was stable with a blood pressure of 110/70 mmHg, absence of neurosensory signs of arterial hypertension, and absence of edema of the lower limbs. At H5 postpartum, the patient presented suddenly and without any clinical signs of the first generalized tonic-clonic seizure, for which immediate treatment was implemented; however, the patient worsened, her neurological condition, with the onset of 4 other generalized tonic-clonic seizures complicated by convulsive status. Faced with worsening symptoms, the parturient was intubated and ventilated and then admitted to intensive care for continued monitoring and surveillance. Note that during convulsive seizures and post-critical, blood pressure monitoring did not identify arterial hypertension or hypertensive peaks.

**Figure 1:**
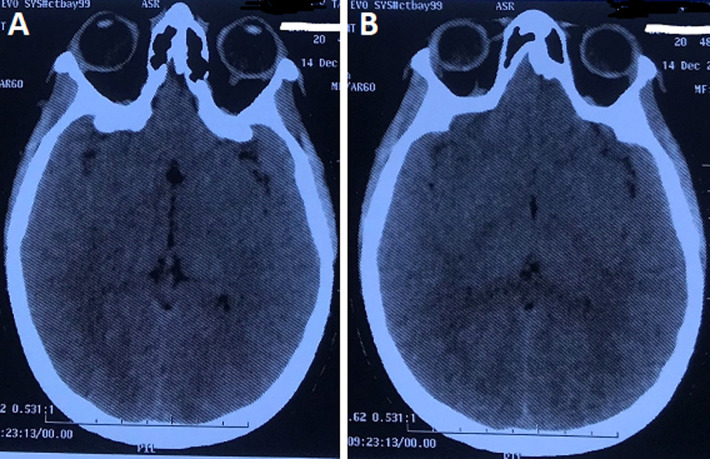
A,B) the brain CT scan showing a cortico - subcortical lateral and posterior asymmetric hypodensities without mass effect with absence of cerebral hemorrhage, which is compatible with a reversible posterior encephalopathy, known as PRES syndrome

**Diagnosis:** the labtix was negative as well as the 24-hour proteinuria: 0.24 mg/24h. A complete biological assessment was carried out and returned to normal: hemoglobin: 11.2 g/dl; platelets: 230,000 elements/mm^3^; urea 0.43 g/l; serum creatinine: 10.64 mg/l; AST: 33 U/L; ALAT: 40 U/L; normal serum electrolytes and prothrombin level at 100%. A pelvic ultrasound was performed showing the visible uterine emptiness line followed to the fundus, no image of retention. The brain CT scan performed showed cortico-subcortical lateral and posterior asymmetric hypodensities without mass effect with the absence of cerebral hemorrhage; the ventricles were of normal morphology. In conclusion: the appearance of the brain CT scan was compatible with a reversible posterior encephalopathy, known as PRES syndrome (Figure 1).

**Treatment:** during the seizures and convulsive state, initial treatment was implemented: conditioning the patient and administration of gardenal and magnesium sulfate (loading dose); faced with worsening symptoms, the parturient was intubated and ventilated and then admitted to intensive care for continued monitoring and surveillance.

**Follow-up and outcome:** the patient remained intubated and ventilated for 48 hours then was extubated, without noticing a recurrence of convulsions. However, the patient was confused for the next 3 days then she showed improvement in her clinical signs and was discharged. After one month, the patient fully recovered.

**Informed consent:** written informed consent was obtained from the patient for participation in our study.

**Patient perspective:** due to the urgent situation, our patient hadn't been aware of the management we had decided. After her recovery, she was informed of her medical situation. She was cooperative and attended all her follow-ups.

## Discussion

The abbreviation PRES stands for Posterior Reversible Encephalopathy Syndrome also known as Posterior Leukoencephalopathy Syndrome (RPLS). This is also called reversible neurological impairment; whose radiological lesions are typically located in the parietal and occipital lobes, hence, the term “posterior” [1]. Its pathophysiology is still poorly understood, but there is a theory that the syndrome is the result of a dysfunction in the autoregulation of cerebral blood pressure which often follows unbalanced hypertension, resulting in a state of neurotoxicity. The latter is manifested by a cerebral edema of the vasogenic type. Another hypothesis implicates an alteration in the permeability power of endothelial cells in the blood vessels of the brain responsible for an accumulation of fluids in the brain tissue [2]. Since the publication of the first case reported in 1996 by Hinchey *et al*. PRES syndrome has always been associated with toxemia of pregnancy, some consider it as a neurological complication of severe preeclampsia, and others go so far as to define it as a radiological expression of eclampsia; seeing the striking clinical resemblance between the two. The incidence of this association continues to increase in view of the widespread use of CT or magnetic resonance imaging (MRI) exploration in the presence of neurological symptoms during pregnancy.

The frequency varies between 86-92% of women with eclampsia and 23% of severe preeclampsia [3,4]. However, and through our case report, we demonstrated an isolated postpartum PRES syndrome without preeclampsia. In recent literature, two atypical cases have been reported; one occurring during pregnancy and the second in postpartum [4,5]. This challenges the theory of causation of the syndrome with preeclampsia-eclampsia and leads us to believe that it is probably a differential diagnosis with eclampsia. A case of PRES syndrome has been reported 3 weeks after the end of chemotherapy treatment for a hydatidiform mole which suggests its relationship with the trophoblast, which requires a uterine revision to check for placental retention when the pathology occurs postpartum [6-8]. Apart from the pregnancy context, there are other etiologies of PRES syndromes, in our patient no particular antecedent was found which could point to another cause of PRES syndrome. Clinically, PRES syndrome shares the same profile with eclampsia that of generalized tonic-clonic seizures often preceded by headache-type prodromes. The convulsions are often repetitive resistant to treatment and lead to a convulsive state as in our case. However, the picture is still not so revealing, in the literature a few cases of atypical clinical presentations which can cause confusion have been reported; such as normal or slightly increased blood pressure, isolated visual disturbances such as sudden onset bilateral blindness or continuous intense headache resistant to treatment suggesting a cerebral aneurysm leading to the performance of a cerebral angiogram [8,9].

The convulsions are often repetitive like in our case where the patient continued to convulse despite the resuscitation measures and the administration of anticonvulsants. CT X-ray images and magnetic resonance imaging of the brain typically reveal a symmetrical cortical and subcortical vasogenic edema on both hemispheres, distributed between the lateral and medial cerebral arterial branches and predominantly parieto-occipital; however, other areas can be affected such as the frontal lobe, temporal lobes, thalami and cerebellum. PRES can also be manifested by diffuse asymmetric partial or extensive cerebral edema, ischemic or hemorrhagic stroke, we can have a mass effect on the cerebral structures or even hydrocephalus, subarachnoid hemorrhage, or a focal hematoma in 15% of cases sometimes then the diagnosis of PRES syndrome becomes difficult [9]. Other radiological manifestations of PRES associated with preeclampsia are more severe vasogenic edema extending to the thalamus and annular protuberance, cerebral hemorrhage and cytotoxic edema. The prognosis is often good, the radiological lesions regress spontaneously within a few days with complete disappearance of the neurological symptoms [9]. But in the literature the syndrome has been linked to a high risk of vasoconstriction and cerebral hemorrhage; despite being said to be reversible, some cases of mortality and neurological sequelae have been observed [9,10].

The following indicators: altered Glasgow score, multiple organ failure, high rate of lactate dehydrogenase (LDH), uric acid, impaired kidney and liver function and thrombocytopenia; represent the risk factors for the aggravation that must be researched and taken into account [10]. Management must be rapid and early in order to avoid the installation of major neurological complications that are difficult to recover, it is based on continuous monitoring of the hemodynamic state, control of blood pressure and the stopping of convulsive seizures. Recently a published study suggests a probable role of hypomagnesemia as a risk factor for PRES syndrome in obstetrics in the acute and chronic phase of the disease, thus proposing magnesium supplementation in pregnant women with PRES syndrome [10].

## Conclusion

Seizures in pregnant women are often linked to eclampsia but, in 12% of cases, other pathologies of cerebral origin may be the cause, such as PRES syndrome. This is why a CT scan should always be performed or MRI especially when the clinical picture is atypical; this attitude will help document neurological pathologies that may coexist with pregnancy without necessarily being associated with preeclampsia.
